# Pronuclear formation by ICSI using chemically activated ovine oocytes and zona pellucida bound sperm

**DOI:** 10.1186/s40104-016-0124-6

**Published:** 2016-11-08

**Authors:** J. E. Hernández-Pichardo, Y. Ducolomb, S. Romo, M. E. Kjelland, R. Fierro, F. Casillas, M. Betancourt

**Affiliations:** 1 División de Ciencias Biológicas y de la Salud, Departamento de Producción Agrícola y Animal, Universidad Autónoma Metropolitana-Xochimilco, Ciudad de México, Mexico; 2 División de Ciencias Biológicas y de la Salud, Departamento de Ciencias de la Salud, Universidad Autónoma Metropolitana-Iztapalapa, San Rafael Atlixco 186, CP 09340 Ciudad de México, Mexico; 3 Departamento de Ciencias Pecuarias, Facultad de Estudios Superiores Cuautitlán, UNAM, Ciudad de México, Estado de México Mexico; 4 Conservation, Genetics & Biotech, LLC, Valley City, ND USA; 5 División de Ciencias Biológicas y de la Salud, Departamento de Biología de la Reproducción, Universidad Autónoma Metropolitana-Iztapalapa, Ciudad de México, Mexico; 6 Doctorado en Biología Experimental, Universidad Autónoma Metropolitana-Iztapalapa, Ciudad de México, Mexico

**Keywords:** Ethanol, ICSI, Oocyte activation, Ovine, Pronucleus, Zona pellucida

## Abstract

**Background:**

In order to improve ICSI, appropiate sperm selection and oocyte activation is necessary. The objective of the present study was to determine the efficiency of fertilization using ICSI with chemically activated ovine oocytes and sperm selected by swim up (SU) or swim up + zona pellucida (SU + ZP) binding.

**Results:**

Experiment 1, 4–20 replicates with total 821 *in vitro* matured oocytes were chemically activated with ethanol, calcium ionophore or ionomycin, to determine oocyte activation (precense of one PN). Treatments showed similar results (54, 47, 42 %, respectively) but statistically differents (*P* < 0.05) than mechanical activated oocytes in sham, ICSI and sham injection (13, 25, 32 %, respectively) (10–17 replicates; *n* = 429).

Experiment 2: Twelve ejaculates and 28 straws of semen were used (11–19 replicates). Sperm were selected by SU in BSA-TCM 199-H medium. A total of 2,294 fresh sperm and 2,760 from frozen-thawed semen were analyzed after SU or SU + ZP binding. Fresh sperm selected by SU showed acrosome reaction (AR) of 59 %, the sperm selected by SU + ZP binding increased AR to 91 %. In comparison, the AR of frozen-thawed sperm using SU or SU + ZP binding was 77 and 86 %, respectively (*P* < 0.05).

Experiment 3: fertilization in 200 mechanical activativated oocytes (17 replicates) was 4 %, but fertilization increased in ethanol activated oocytes after ICSI (12-28 %) (5–6 replicates). When fresh sperm only selected by SU were injected to 123 oocytes, a fertilization rate (28 %) was achieved; in sperm selected by SU + ZP was 25 % (73 oocytes). In comparison, in frozen-thawed sperm selected by SU, fertilization was 13 % (70 oocytes), whereas sperm from SU + ZP binding displayed 12 % (51 oocytes) (*P* > 0.05).

**Conclusions:**

Chemical activation induces higher ovine oocyte activation than mechanical activation. Ethanol slightly displays higher oocyte activation than calcium ionophore and ionomicine. Sperm selection with SU + ZP increased AR/A and AR/D rates in comparison with SU in fresh and frozen-thawed sperm. According to this, in terms of fertilization rates, chemical activation after ICSI increased oocyte PN formation compared to mechanical activation. Also, fresh sperm treated with SU and SU + ZP were significantly different than frozen-thawed sperm, but between sperm treatments no significant differences were obtained.

## Background

In recent decades, assisted reproductive techniques (ARTs) in mammals, have been developed for its use in superovulation, embryo transfer (ET), *in vitro* fertilization (IVF), intracytoplasmic sperm injection (ICSI) and cloning by somatic cell nuclear transfer. Despite the great advances in the protocols of these ARTs, their efficiency still remains low [1].

Among ARTs, ICSI is the current procedure for avoiding polyspermy and increasing the number of produced zygotes, as well as allowing for the study of several aspects of fertilization such as gamete interaction, sperm decondensation, oocyte activation [2] and gene transfer [3, 4]. In human, this technique has been developed with great success [5]; however, its application in livestock is still not well established, mainly due to the different reproductive physiology among species of economically important domestic animals such as cattle, pigs, horses and sheep. In general, embryo development (ED) using the ICSI procedure is lower than that of IVF [2, 6].

Although ovine oocyte fertilization by means of the injection of immobilized sperm has been done before, and normal lambs have been born [7, 8], ICSI success is markedly restricted due to sperm head descondensation failure and low male pronuclei (PN) formation [9]. In turn, several studies have tried to solve this issue in sheep using sperm pretreatments before performing ICSI. In order to induce sperm head decondensation and male PN formation, sperm have been pre-treated with lysophosphatidylcholine (LPC) [10], triton X-100 [11], dithiothreitol (DTT), sodium dodecyl sulphate (SDS) or by freezing and thawing without cryoprotectants [9]. These treatments are directed at removing the sperm membranes and dissolving the nuclear proteins allowing the head decondensation.

In human, physiological strategies are being explored to investigate the selection of sperm that are more capable of achieving successful ICSI, compared to the subjective way that the embryologists currently use [12], increasing fertilization. A strategy for improving ICSI is using sperm attached to the zona pellucida (ZP) [13, 14]. Studies [12, 14, 15] have concluded that the ZP has the ability to select functionally normal sperm and even those with a superior quality. Further, the attachment to the ZP during fertilization is necessary for inducing the acrosome reaction (AR), which is an important factor for ICSI success.

Other important factor is the oocyte activation which is the resumption of meiosis II during fertilization increasing calcium ion levels in the cytoplasm. Without this step, the inseminated oocyte prevents the decondensation of the sperm head. Notably, artificial stimuli can mimic the action of sperm during fertilization. Chemical activation of oocytes has been necessary with ICSI because mechanical activation alone, by means of sperm injection, is sometimes not sufficient for inducing oocyte activation [16, 17]. To improve results, appropriate sperm treatment and oocyte activation is necessary to achieve fertilization [17].

Oocyte activation has not been shown to be crucial in several species such as mice, hamsters and rabbits; however, it is essential in pigs, cattle and sheep [18]. The chemical activation of ovine oocytes after ICSI has been performed using: ionomycin (ION) [17, 19, 20], ION+ 6 dimethylaminopurine (6-DMAP) [4, 9, 17, 21, 22], ethanol (ETL) [4, 21], DTT [9] and calcium ionophore (CAI) [16]. However, even with such chemical treatments, the percent of sheep blastocysts produced after ICSI and cultured *in vitro* was around 20 %. Moreover most investigations have evaluated ED but not fertilization, determined by PN formation, to assure that the zygotes are diploid. This condition is essential for the normal development of the embryos [23]. When oocytes are artificially activated, a high probability that the resultant blastocysts will be parthenogenic exists [17, 24, 25]. Therefore, the objective of the present study was to determine the efficiency of fertilization using ICSI with chemically activated ovine oocytes with sperm selected by swim-up (SU) or SU plus binding to ZP (SU + ZP).

## Methods

The ovaries were collected from a slaughterhouse, “El Rojo”. The aforementioned facility has the animal health federal law authorization under the number 150810056630. Except for the maturation and development media, which were prepared in a commercial laboratory (In Vitro S.A., México), all chemicals were purchased from Sigma Chemical Company (ST Louis, MO USA). All incubation conditions were performed at 38.5 °C in an atmosphere with 5 % CO_2_, 95 % air, and humidity at saturation (NUAIRE, USA). *In vitro* maturation (IVM) and ICSI procedures were performed under mineral oil (Fisher Scientific, USA). All media were stored at 4 °C for not more than three wk and supplemented 24 to 48 h before use.

### Experimental design

The experimental design consisted of three separate experiments. Experiment 1 was conducted to identify the percent activation of oocytes matured *in vitro* only exposed to chemical activation with ETL (*n* = 393), CAI (*n* = 350), ION (*n* = 78) or mechanical activation through ICSI (*n* = 200), sham injection (false injection) (*n* = 78) or sham (only manipulation of the gametes without injection) (*n* = 151), and evaluated by the PN formation.

Experiment 2 was conducted to determine the viability and sperm acrosome status after selection by SU or SU + ZP of fresh (*n* = 1,194, SU; *n* = 1,100 SU + ZP) and frozen-thawed sperm (*n* = 718, SU; *n* = 922 SU + ZP).

Experiment 3 was conducted to determine fertilization evaluated by the formation of two PN and without the presence of a sperm head when performing ICSI with fresh or frozen-thawed sperm selected by SU or SU + ZP. After ICSI, oocyte chemical activation with 7 % ETL for 5 min (*n* = 123 SU; *n* = 73 SU + ZP and *n* = 70 SU; *n* = 51 SU + ZP respectively) and mechanical activation (*n* = 200) were performed.

### Oocyte collection and maturation

Ovaries were collected from adult ewes and transported, less than three h, to the laboratory in 0.157 mol/L NaCl solution at 37 °C with antibiotics (10,000 UI/mL ampycillin, 10,000 μg/mL streptomycin and 25 μg/mL, amphotericyn (In Vitro S.A., México). Ovaries were rinsed three times in the fresh solution described above. Follicular fluid was aspirated from 2 to 6 mm follicles punctured with an 18-gauge needle fixed to a 10 mL disposable syringe containing 1 mL of modified Tyrode’s medium supplemented with 10 mmol/L sodium lactate, 10 mmol/L HEPES and 0.1 % polyvinilic alcohol (PVA) (TL-HEPES-PVA) with a pH of 7.3–7.4, supplemented with 200 UI/mL heparine. Follicular fluid was pooled in a 50 mL conical tube (PISA, México). Cumulus cell-oocyte complexes (COCs) with compact cumulus mass and oocytes with uniform cytoplasm were selected and washed three times in 500 μL drops of maturation medium TCM-199 with Earle’s salts, 26.2 mmol/L sodium bicarbonate and L glutamine (In Vitro S.A., México), supplemented with 0.1 % PVA, 3.05 mmol/L D-glucose, 0.91 mmol/L sodium pyruvate, 0.57 mmol/L cysteine and 10 ng/mL epidermal growth factor [26].

For oocyte maturation, sterile four well plates were used (Thermo-Scientific Nunc, Rochester NY). First, 500 μL of maturation medium with 0.5 μg/mL LH, 0.5 μg/mL FSH and 10 % (v/v) fetal calf serum (FBS) (In Vitro S.A., México) was added to each well. Next, 20–30 COCs were placed in the well with the maturation medium and incubated for 24 h [19]. After IVM, COCs were placed in 500 μL of TCM 199-HEPES (TCM 199-H) with 300 IU of hyaluronidase for three min, then washed three times in TCM 199-H [13]. Maturation was determined by identification of the first polar body (PB) using an inverted microscope (Nikon Eclipse TE200, Japan).

### Oocyte activation

For experiment 1, mature denuded oocytes (only with a PB) were distributed randomly among the following groups to perform activation; mechanical activation: (1) Sham: oocytes manipulated as in ICSI without injection, (2) ICSI, (3) Sham injection: oocyte pierced without sperm insertion, and for chemical activation without sperm injection: (4) 7 % ETL for 5 min, (5) 50 μmol/L CAI for 10 min, and (6) 5 μmol/L ION for 5 min. After activation, oocytes were washed three times in modified Tris-buffered Medium (mTBM) consisting of 13.1 mmol/L NaCl, 3 mmol/L KCl, 7.5 mmol/L CaCl_2_ · 2H_2_O, 20 mmol/L Tris, 11 mmol/L glucose, 5 mmol/L sodium pyruvate, supplemented with 0.4 % bovine serum albumin (BSA) fraction V, and 2.5 mmol/L caffeine benzoate and placed in 200 mL of medium and incubated during 18 h.

### Assessment of pronucleus formation

After incubation, oocytes were stained with 10 μg/mL bisbenzimide (Hoechst 33258) in cold ETL for 15 min, protected from the light. Further, oocytes were placed on a slide in a microdrop of PBS-glycerol (1:9) [9], and examined under an epifluorescence microscope at 400× (Eclipse E600, Nikon, Japan).

Non-activated oocytes were determined by the identification of the metaphase II, whereas activated oocytes presented a PN.

### Sperm preparation

Semen was collected from a creole ram of proven fertility, located in the animal facilities at the Universidad Autónoma Metropolitana-Xochimilco, as approved under the regulations of the Committee for Care and Use of Animals. One hundred μL of fresh or frozen semen were added to 2 mL of TCM 199-H supplemented with 3 mg/mL BSA (BSA-TCM 199-H), and incubated for 1 h for SU to help capacitation. Afterwards, 500 μL of medium were gently removed from the top of the suspension, then 3 mL of BSA-TCM 199-H were added and centrifuged at 200 ×g for 10 min [9, 27]. The pellet was suspended to give a final concentration of 1×10^6^ sperm/mL [28].

### Exposure of sperm to mature oocytes ZP

In each assay, four oocytes in metaphase II were placed in 400 μL drops of BSA-TCM-H and 1 % gentamycin, followed by the addition of 100 μL of the sperm suspension and incubated for 1 h. The oocytes were transferred to a 300 μL drop of TCM-H medium and washed by repeated aspirations with a fine glass pipette to eliminate the non- bound ZP sperm. Two oocytes with sperm tightly bound to the ZP were placed in 20 μL drops of the same medium and covered with mineral oil. The sperm bound to the ZP were removed by repeated aspiration of the oocytes through a fine glass pipette, for ICSI [13, 29], while the other two oocytes were placed in 100 μL drops to obtain the sperm for viability and AR analysis.

### Viability and acrosomal status of sperm after SU and ZP binding

Fresh and frozen-thawed sperm were selected by SU or SU + ZP binding. To determine their viability and acrosome status, propidium iodide (PI) and fluorescein isothiocyanate with peanut agglutinin (FITC-PNA) double staining was developed [30, 31]. From each sperm suspension sample, 100 μL were obtained. Next, 5 μL of FITC-PNA and 5 μL of PI were added, followed by incubation for 5 min. Ten μL of the sperm suspension were mixed with 10 μL 1.6 % paraformaldehyde in a slide and analyzed under a fluorescence microscope (Eclipse E600, Nikon, Japan) at 400×. The functional status of the sperm was classified according to the following staining patterns: live sperm with intact acrosome: cells with FITC-PNA stain at the acrosome, without PI stain at the post-acrosomal region. Live sperm with AR: cells without FITC-PNA acrosome stain and without PI nuclear stain. Dead sperm with intact acrosome: cells with FITC-PNA stain in the acrosome region, and with PI nuclear stain. Dead sperm with AR: without FITC-PNA stain in the acrosome region, and with PI nuclear stain.

### Intracytoplasmic sperm injection

For ICSI, a 50 μL drop of TCM 199-H, supplemented with 2 % FBS and 1 % gentamycin containing five *in vitro* matured denuded oocytes, and one 10 μL drop of sperm suspension with 10 % polivinilpirrolidine (PVP) (1:1 dilution) were added to a 6 cm diameter Petri dish [8, 16]. Microinjection was performed as previously reported by Shirazi et al. [9], using an inverted microscope at 200× (Nikon Eclipse TE200, Japan). The microinjecton pipette was immersed in the sperm suspension-PVP and one sperm was immobilized by scoring the midpiece and aspirating the tail-first, and then injected into the ooplasma through the ZP. The first PB was either in the 6 or 12 o’clock position, and the injection pipette was in the 3 o’clock position. During the injection, cytoplasm was aspirated to ensure that the oolema was punctured. After ICSI, oocytes were washed three times in BSA-TCM 199-H. Mechanical and chemical activated oocytes were incubated in 100 μL of mTBM for 18 h. A total of 517 oocytes were injected (5–17 replicates).

### Oocyte chemical activation after ICSI

For experiment 3, 10–20 ICSI inseminated oocytes were activated in 100 μL drops of 7 % ETL (Baker Absolute alcohol 9000-02) in TCM 199-H at room temperature for 5 min [32]. After activation, oocytes were washed and incubated in mTBM during 18 h.

### Nuclear staining

Eighteen h after ICSI, oocytes were stained with 10 μg/mL Hoechst 33258 as mentioned, to determine the following stages: 1) non-activated: oocytes in metaphase II and without a decondensed sperm head; 2) activated oocytes: formation of the female PN and without a decondensed sperm head; and 3) fertilized oocytes: oocytes with two PN and without the presence of the sperm head [9, 19, 33].

### Statistical analysis

With regard to the oocyte activation, 4–20 replicates were performed. For sperm viability and acrosome status, 11–19 replicates were performed for fresh and frozen-thawed semen. To assess fertilization 5–6 replicates were performed for each group. ANOVA and Bonferroni multiple post hoc test were used for comparing the results of the following variables: oocyte activation, viability and acrosome status of fresh sperm compared to frozen-thawed sperm, and fertilization with mechanical and chemical activation, but also between sperm treatments [34], considering significant differences between the groups when *P* < 0.05.

## Results

### Oocyte activation

The mechanical activation of oocytes with sham injection was higher than sham (*P* < 0.05). However, no differences were observed between sham injection and ICSI. Chemical activation with ETL, CAI and ION was higher than the mechanical activation (*P* < 0.05). When compared to chemical activation with ETL, the three chemical activation treatments had a similar proportion of PN with a slightly increase in the ETL group, shown in Table 1. Based on these results, oocytes subjected to ICSI were hereafter treated only with 7 % ETL for 5 min.

**Table 1:** Activation procedures in sheep oocytes matured *in vitro*

	Mechanical activation			Chemical activation		
	Sham	ICSI	Sham Injection	ETL	CAI	ION
N	13	17	10	20	19	4
n	151	200	78	393	350	78
Activated oocytes,%	2,0 13 ± 1.6^a^	4,9 25 ± 0.5^ab^	25 32 ± 3.4^b^	21,1 54 ± 1.3^c^	16,4 47 ± 1.3^c^	3,3 42 ± 5.0^c^

Sham: oocytes manipulated as in ICSI but no injection; Sham injection: oocyte pierced but no sperm insertion; ETL: 7 % Ethanol 5 min; CAI: 50 μmol/L Calcium Ionophore 10 min; ION: 5 μmol/L Ionomicine 5 min. Mechanically activated oocytes: Oocytes in metaphase II. Activated oocytes: Presence of one pronucleus

*N* number of replicates; *n* number of oocytes examined

Percentage data are presented as mean ± SD

^a,b,c^Values with different letters indicate statistically significant differences (*P* < 0.05)

### Viability and acrosome reaction

Independently of whether the sperm were alive or dead, the proportion with AR was greater in those selected with SU + ZP than SU only (control) (*P* < 0.05), either in fresh or frozen-thawed semen. These results indicate that if ICSI is performed using sperm selection through ZP binding, the probability of injecting sperm with AR is very high, independently of the viability and fresh or frozen-thawed condition, shown in Table 2.

**Table 2:** Viability and acrosome status of sperm selected by swim-up or swim-up and oocyte ZP binding

		Fresh Semen, %					Frozen-thawed semen, %			
		AR/A	AR/D	NAR/A	NAR/D		AR/A	AR/D	NAR/A	NAR/D
SU	*N* = 12					*N* = 14				
	*n* = 1,194	52,8 44 ± 1.8^a^	17,9 15 ± 0.8^a^	34,0 28 ± 8.3^a^	14,7 12 ± 0.6^a^	*n* = 1410	71,8 51 ± 0.9^a*^	37,3 26 ± 0.9^a*^	17,5 12 ± 0.3^a*^	14,4 10 ± 0.5^a^
SU + ZP	*N* = 11					*N* = 19				
	*n* = 1,100	64,8 59 ± 0.9^b^	34,7 32 ± 1.1^b^	40 4 ± 0.3^b^	65 6 ± 0.2^b^	*n* = 1350	92,2 68 ± 1.8^b*^	23,7 18 ± 0.9^b*^	14,3 11 ± 0.5^a*^	48 4 ± 0.2^b*^

*SU* swim-up, *ZP* Zona pellucida, *AR/A* acrosome reaction/alive, *AR/D* acrosome reaction/dead, *NAR/A* no acrosome reaction/alive, *NAR/D* no acrosome reaction/dead, *N* number of replicates, *n* number of sperm examined

Percentage data are presented as mean ± SD

^a,b^Values in the same column with different letters are significantly different (*P* < 0.05)

*Within rows, among the variables corresponding to fresh and frozen-thawed semen counterparts, significant difference was observed (*P <* 0.05)

### ICSI

The oocytes treated with ETL had an increased fertilization rate with ICSI (presence of two PN without the sperm head) compared to mechanical activation. Fertilization was increased when fresh semen was used compared to frozen-thawed semen, independent of the sperm selection procedure (*P <* 0.05). The comparison of the four groups of oocytes exposed to chemical activation with ETL were significant different than mechanical activation (*P* < 0.05), shown in Table 3. Also, a significant difference in fertilization was observed between the fresh and frozen-thawed sperm but between selection (SU and SU + ZP) no significant differences were obtained (Table 3). The percentage of activated oocytes was greater than percentage of fertilized oocytes, indicating that the proportion of sperm decondensation is still lower when the oocytes are activated.

**Table 3:** Effect of oocyte activation, semen type and sperm selection technique for ICSI on fertilization percentage

Semen type	Frozen-thawed semen	Fresh		Frozen-thawed	
Oocyte treatment	Mechanical activation	Activated with 7 % Ethanol			
Sperm Treatment	“Swim-up”	“Swim-up”	“Swim-up” + Zona pellucida	“Swim-up”	“Swim-up” + Zona pellucida
N	17	5	6	5	5
n	200	123	73	70	51
Activated ooctyes, %	4,9 25 ± 2.7	7,5 61 ± 1.3*	3,6 49 ± 1.6*	4,0 57 ± 3.2*	3,1 61 ± 6.3*
Fertilized ooyctes, %	8 4 ± 0.2	3,5 28 ± 1.7*	18 25 ± 1.7*	9 13 ± 1.2*^▲^	6 12 ± 0.9^▲^

Activated oocytes: Formation of female pronucleus and no decondensed sperm head; Fertilized oocytes: Oocytes with two pronuclei without sperm head

*N* number of replicates, *n* Number of oocytes examined

Percentage data are presented as mean ± SD

*Indicates significant differences with respect to mechanical activated oocytes (*P* < 0.05)

^▲^Indicates significant differences with respect to SU fresh (*P* < 0.05)

## Discussion

The chemical activation of the sheep oocytes during ICSI is necessary due to the low percentages of sperm decondensation and PN formation. Oocyte activation implies an increase of intracellular free calcium, causing the degradation of the maturation promoting factor (MPF), which is essential for the resumption of the second meiotic division and the formation of both PN [25, 35].

ETL is among the most utilized chemical activators through the formation of inositol 1,4,5-triphosphate and its union to the membrane receptors in the endoplasmic reticulum releasing of intracellular calcium [25, 36, 37]. Other chemical activators include CAI and ION, which induce the liberation of intracellular calcium stored in the endoplasmic reticulum, evident by the activation of several proteolytic pathways calcium dependent that drive the destruction of cyclin B, reducing the activity of MPF [25, 36, 38].

In regard to experiment 1 the present study demonstrated that chemical activation induced a higher female PN formation than that produced by mechanical activation. The results obtained with ETL oocyte activation were similar to those reported by Shirazi et al. (50 %) [36] who studied the effect of sheep oocyte maturation on parthenogenetic development. In other mammalian domestic species, oocyte activation with ETL generally leads to a lower incidence of pronuclear formation. Despite this, Wang et al. [39] activated cow oocytes and reported 42 % activation. Additionally, Yi and Park [40] observed an increase in the activation of pig oocytes with ETL exposure (34 %).

Also, in the present study, the use of CAI resulted in an oocyte activation of 47 %. However, little is known about sheep CAI oocyte activation. Nevertheless, studies in cattle reported 22 % [39]. Oocyte activation using ION resulted in 42 % PN formation, which is similar to that reported by Shirazi et al. [36], who evaluated activation through ED. These results support the use of these chemical activators with sheep oocytes, which are essential in ICSI.

In the present study, mechanical activation by ICSI displayed 25 % PN formation, similar to that reported by Gómez et al. [8, 33] with 31 % (determined through ED) and 38 % (resumption of second meiotic division), while Shirazi et al. [17] observed a PN formation of 5 % only. The effect of the sham injection resulted in 32 % of the oocytes undergoing PN formation. The results of this study are superior to those reported by Gómez et al. [10], with 22 %.

The sham group showed that 13 % of the oocytes were activated, which is similar to those by Catt and Rhodes [41]. This could be due to the use of hyluronidase during the removal of cumulus cells, since this enzyme is capable of producing oocyte activation in mice, cattle and human itself [32]. With regard to the activation using sham injection or ICSI, our results indicate that the injection by itself induces sheep oocyte activation and not necessarily the presence of a sperm.

Several factors influence oocyte sensitivity to artificial activation and this can explain the differences between the results obtained in the present study and those reported previously. Factors influencing oocyte activation include: differences among species [37], *in vivo* or *in vitro* maturation, presence or absence of cumulus cells [32, 36] and type of stimuli [32].

For improving ICSI results, new methods of sperm selection are being developed in human, as injecting sperm previously bound to the ZP of oocytes. To our knowledge, this is the first study that evaluates the acrosome status and viability of sperm from fresh and frozen-thawed ovine sperm after being in contact with the *in vitro* matured oocyte ZP of the same species. It is important to consider that the use of ZP does not just select sperm, but also induces AR, which has been reported to be essential for decondensation of sperm chomatin in ooplasm [9]. In experiment 2 the percentage of sperm with AR after the ZP binding was 91 and 86 % for fresh and frozen-thawed sperm, respectively, which are similar to those reported with fresh goats semen, [42] pigs [43] and human [44]. Notably, a significant difference was observed between the SU + ZP and SU selection procedures. Among the factors that affect the trigger of the AR through ZP contact, is the state of oocyte maturation; immature porcine oocytes in contact with fresh semen for 10 min had an AR of 34 %, while mature oocytes 42 % underwent AR, similar tendency was observed when frozen-thawed sperm was used with immature (37 %) and mature oocytes (45 %) [45].

In experiment 3, a significant increase in the percentage of activated and fertilized oocytes was observed when stimulated with ETL after ICSI, similar results were obtained by Shirazi et al. [17], besides, they reported that mechanical stimulation (ICSI or sham injection) is not sufficient to activate ovine oocytes for PN formation, indicating that chemical activation is essential for ICSI in sheep.

Oocyte treatment with ETL after ICSI increased the oocyte activation percentage from 49 to 61 %, and 12 to 28 % for fertilization, compared to mechanical activated (25 and 4 %, respectively). The fertilization rate with fresh sperm was only 50 % with respect to the proportion of the activated oocytes; while that in frozen-thawed sperm was not higher than 25 %. If the evaluation of these oocytes was through the ED, more than 50 % could be parthenotes. Similar results have been reported in cattle oocytes treated with ETL after ICSI with frozen-thawed sperm heads, with 52 % of activation, but only 27 % of fertilization, being 56 % parthenote embryos [46]. In equine oocytes with the same treatment, 56 % activation, 16 % fertilization, and 40 % parthenotes have been obtained; indicating that even when oocytes are activated, the sperm decondensation may not occur, but artificial activation can induce parthenogenetic development in oocytes with ICSI [47].

In the present study, an increase in fertilization was not observed using sperm previously bound to oocyte ZP and with an AR higher than 85 %. This finding is in agreement with that reported by Gómez et al. [10], in which an increase in fertilization was not observed (22 %) after ICSI with AR sperm (86 %) compared to that obtained with untreated sperm (33 %). These results indicate that there is not a correlation between the percentage of sperm with AR and the number of oocytes fertilized, suggesting that it is not necessary to induce AR before ICSI in sheep to obtain fertilization. In human, Black et al. [13] did not observe a significant difference in fertilization between ICSI performed with sperm bound to the ZP and conventional ICSI; however, higher quality embryos and a greater number of pregnancies were produced.

A significant difference in the fertilization rate was observed between oocytes fertilized with frozen-thawed and fresh sperm. This is consistent with Gómez et al. [10], reporting higher fertilization rates after ICSI with fresh sperm than frozen-thawed sperm. Also Gómez et al. [10] results could be due to acrosome-membrane damage which may occur during the freeze-thaw process; thereby predisposing sperm to further membrane changes or some other deleterious effect on sperm DNA. In addition, in human sperm, it has been reported that the freeze-thaw process produces an oxidative stress due to the generation of oxygen free radicals and an alteration of the sperm antioxidant system [48]. The aforementioned oxidative stress can cause the peroxidation of membrane lipids, alterations of proteins and DNA fragmentation [49] which decreases sperm fertilization capacity.

## Conclusions

Chemical activation induces higher ovine oocyte activation than mechanical activation. ETL slightly displays higher oocyte activation than CAI and ION. Sperm selection with SU + ZP increased AR/A and AR/D rates in comparison with SU in fresh and frozen-thawed sperm. According to this, in terms of fertilization rates, chemical activation after ICSI increased oocyte PN formation compared to mechanical activation. Fresh sperm selected by SU and SU + ZP were significantly different than frozen-thawed sperm, but between both selection procedures no differences were obtained. Therefore, this study demonstrates that chemical activation is recquired to activate oocytes after injection and acrosome reaction is not essential for fertilization. In turn, fresh sperm was more efficient in terms of fertilization capacity when performing ICSI.
